# Comparison of High-Intensity Laser Therapy and Ultrasound Treatment in the Patients with Lumbar Discopathy

**DOI:** 10.1155/2015/304328

**Published:** 2015-03-25

**Authors:** Ismail Boyraz, Ahmet Yildiz, Bunyamin Koc, Hakan Sarman

**Affiliations:** ^1^Department of Physical Medicine and Rehabilitation Training and Research Hospital, Abant Izzet Baysal University Medical School, Bolu, Turkey; ^2^Department of Orthopeadic and Traumatology, Abant Izzet Baysal University Medical School, Bolu, Turkey

## Abstract

The aim of the present study was to evaluate the efficiency of high intensity laser and ultrasound therapy in patients who were diagnosed with lumbar disc herniation and who were capable of performing physical exercises. 65 patients diagnosed with lumbar disc were included in the study. The patients were randomly divided into three groups: Group 1 received 10 sessions of high intensity laser to the lumbar region, Group 2 received 10 sessions of ultrasound, and Group 3 received medical therapy for 10 days and isometric lumbar exercises. The efficacy of the treatment modalities was compared with the assessment of the patients before the therapy at the end of the therapy, and in third month after the therapy. Comparing the changes between groups, statically significant difference was observed in MH (mental health) parameter before treatment between Groups 1 and 2 and in MH parameter and VAS score in third month of the therapy between Groups 2 and 3. However, the evaluation of the patients after ten days of treatment did not show significant differences between the groups compared to baseline values. We found that HILT, ultrasound, and exercise were efficient therapies for lumbar discopathy but HILT and ultrasound had longer effect on some parameters.

## 1. Introduction

The lumbar region is the most common site involved in musculoskeletal pain. In developed countries, low-back pain ranks second after headaches among the other causes of pain. Of people living in industrialized countries, approximately 80% suffer from low-back pain at a certain time in their lives [1]. Approximately 10% of people who experience low-back pain develop chronic low-back pain. Approximately 1% of the population is completely disabled due to low-back pain. Low-back pain often starts at a young age, and the prevalence is the highest in middle-aged population [1]. Intervertebral disc diseases, which are an important etiological cause of low-back pain, often occur in the lumbar region (61.94%). The majority of people presenting with low-back pain have problems with intervertebral discs. There are many different approaches in the management of low-back pain. There is a wide spectrum of treatment options including patient education, behavioral therapies, lumbar support, and physical therapy modalities such as massage, traction, superficial heaters, deep heaters, transcutaneous electrical nerve stimulation (TENS), and laser. The treatment of disc herniation is important to control pain, to prevent the recurrence, and development of chronic pain and disability, and to accelerate the return to work process. Exercises and education on lumbar protective measures have become prominent in recent years [2].

The term laser originated as an acronym for “light amplification by stimulated emission of radiation.” The basic principle of laser devices is the amplification of electron spin rates by passing photon energy through a particular medium to produce a single directional laser beam having a different wavelength than the original light beam [3]. The action mechanism of lasers is based on tissue stimulation. This stimulation occurs at the level of the cell, vascular structure, interstitial tissue, and immune system. Furthermore, laser has direct effects when applied to the tissues locally and systemic effects when applied to acupuncture points [4]. The analgesic and anti-inflammatory effects of laser can be explained by many mechanisms. Laser produces reactive vasodilation by decreasing the pain sensation in the sensory nerve endings and the spasm in the muscle arterioles. It exerts analgesic and anti-inflammatory effects by promoting regeneration and increasing the release of beta-endorphins through the induction of protein synthesis in the rheumatoid synovial fluid. Laser is also suggested to stimulate hematopoiesis in the bone marrow and exert antibacterial effects by stimulating the immune system [4]. Lasers do not cause a significant change in the tissue temperature. This finding indicates that the potential physiological effects of laser are independent from heat. Recent studies implicated laser in the regenerative process of the tissue, bone formation, synthesis of new cartilage tissue, and synthesis of the cartilage matrix [5, 6]. It was found that Nd: YAG lasers contribute to the healing process in the tendons and ligaments and prevent the formation of fibrosis [7]. Some studies showed that low level laser therapy combined with exercise had more beneficial than exercise alone in chronic low-back pain for the long term [8–10].

Superficial and deep heaters used in the treatment of lumbar disc herniations have an important place in physical therapy applications. Superficial and deep heaters have multiple effects such as vasodilation, increased pain threshold, and increased collagen production in connective tissues. It was found that ultrasound (US) exerts many effects mediated by its thermal effects such as increase in nerve transmission speed and enzymatic activity, increase in the contractility of skeletal muscles, increase in the elongation of collagen tissue, increase in blood flow rate, decline in pain threshold, and relief of muscle spasms [11]. US is important physical therapy agent used in the treatment of musculoskeletal disorders [12].

The aim of the present study is to evaluate the efficiency of high intensity laser and ultrasound therapy in patients who are diagnosed with lumbar disc herniation and who are capable of performing physical exercises.

## 2. Materials and Methods

The present study included patients who were admitted to the outpatient or inpatient clinic of Physical Therapy and Rehabilitation at our hospital to undergo a physical therapy program and who met the study inclusion criteria. The diagnoses of the patients were established by medical history, physical examination, and results of imaging studies. The diagnose of 65 patients confirmed with lumbar MRI as lumbar disc herniation. The patients were randomly divided into three groups: Group 1 received 10 sessions of high intensity laser to the lumbar region five sessions per week, Group 2 received 10 sessions of US to the lumbar region five sessions per week, and Group 3 received medical therapy (NSAII) for 10 days and all of the patients in three groups performed isometric lumbar exercises. The efficacy of the treatment modalities was compared with the assessment of the patients before the therapy, at the end of the therapy, and in third month after the therapy.

The patients, who were diagnosed with lumbar disc herniation on lumbar MRI performed, who were not working on occupations requiring intensive effort and in whom physical therapy was not contraindicated, who did not have congenital abnormalities or history of trauma, and who had sufficient mental capacity to understand and answer the questions asked in the assessment scales, were included in the study. The patients who had a history of injection to the lumbar region in the last four weeks or who had severe osteoporosis, history of lumbar surgery, acute trauma, inflammatory pain, neurological disorder, or lumbar instability, patients who received physical therapy in the last three months, and patients with uncontrolled or severe cardiovascular or metabolic disorder were excluded from the study.

A detailed medical history was obtained from the patients and all underwent physical examination of the locomotor system. The patients were randomly divided into three groups: Group 1 included 20 patients, Group 2 included 25 patients, and Group 3 included 20 patients. VAS (visual analog scale) was used to assess the pain level of the patients. The Oswestry disability index, SF-36 (short form 36), was used to evaluate the functional and psychologic status of the patients. A locomotor system examination was repeated after the therapy.

The patients in Group 1 received laser therapy 3.8 watts for 14 minutes at a wavelength of 1064 nm. The total energy received was 1800 joules. A cosmogamma Cyborg laser device was used as the high intensity laser in this study. This device produces laser beams with a wavelength of 1064 nm. This device is also known as a gallium aluminum arsenide laser (GaAlAs laser) and designed to provide a fiber output of at least 10 w (±10%). The device has continuous, pulsed, and high pulsed modes. Different treatment programs are recorded on the device memory according to different diagnoses. The treatments were applied to the lumbar region using beam expanders for the treatment of large areas up to 120 cm^2^.

The patients in Group 2 received a US therapy. A Chattanooga intelect mobile US device was used in the treatments. The intelect mobile US device allows the application of 1 or 3 MHz, and 20% or 50% or continuous modes without any need to change the applicators. In the present study, US was applied at 1.5 watt/cm for six minutes to the lumbar paravertebral area. In addition, an isometric lumbar exercise program was initiated to be performed with five repetitions in each set (modified straightening and pelvic tilt exercises) in Groups 1 and 2. The repetitions of both sets were increased up to ten, provided that this did not increase the patient's pain.

The patients in Group 3 received a medical therapy agent for ten days in addition to two sets of lumbar isometric exercises (pelvic tilt and modified straightening), which were repeated five times in the morning and at night. All patients in the study were trained on lumbar exercises. The patients were administered pelvic tilt and modified straightening exercises, to be performed in two sets, each containing at least five repetitions during periods with intensive pain. The patients were instructed to increase the number of repetitions to ten in each set when the treatment provided some relief. The patients were informed that the key to prevent recurrences and provide functional recovery was making the exercises part of their lives.

The demographic features of the patients were questioned. The patient's age, place of residence, comorbid conditions, and medications were questioned. The patients were assessed before and after the therapy. The lumbar MR images of the patients were evaluated.

*Statistical Analysis.* All statistical analyses were performed using SPSS 17.0 for Windows (SPSS, Chicago, IL, USA). The Kolmogorov-Smirnov test was used to test the normality of the data distribution, and the data were expressed as the mean and standard deviation. The chi-square test was used to compare the categorical variables between the groups. The one-way ANOVA test was used for comparisons of the parametric continuous data. The Kruskal-Wallis test was used for the nonparametric continuous data. Pearson's correlation analysis was used to examine the associations between the variables, and a linear regression analysis was performed to identify independent predictors of the pain domains of the SF-36. A two-sided *P* value < 0.05 was considered to be statistically significant. A repeated measures ANOVA was used to analyze the changes in variables. Significant differences were determined by Bonferroni post hoc tests.

## 3. Results

Of 20 patients in Group 1, 5 were males and 15 were females. Of 25 patients in Group 2, 8 were males and 17 were females. Of 20 patients in Group 3, 9 were males and 11 were females. There was no statistically significant difference in terms of gender distribution. The mean age was 58.4 ± 10.76 years in Group 1, 61 ± 10.47 years in Group 2, and 54.6 ± 14.89 years in Group 3. There was no significant difference between the groups in terms of age (*P* > 0.05).

The lumbar MRI reports of 65 patients with lumbar disc herniations were examined. Of these patients, 53 had disc protrusion at one or more levels and 12 had disc extrusion. Of 65 patients, 32 had compression of the nerve roots at one or more levels. There was no significant difference in terms of compression of the nerve root and level of disc herniation.

The comparison of parameters in Group 1 before the treatment and at the end of the therapy revealed significant changes in VAS (visual analog scale), Oswestry scale score, BP (body pain), GH (general health), VT (vitality), and SF (social functioning) (*P* < 0.05). There was no significant change in PF (Physical Function), RP (Restricted Physical Roles), RE (Restricted Emotional roles), and MH (Mental Health) parameters (*P* > 0.05). Changes of Oswestry scale score and PF, BP, GH, and VT parameters in third month after the therapy compared to values at the end of the treatment were statically significant (Table 1).

The comparison of parameters in Group 2 before the therapy and at the end of the therapy revealed significant changes in VAS score, Oswestry scale score, and PF, RF, BP, GH, VT, SF, RE, and MH parameters (*P* < 0.05). Comparing results in third month of the treatment and at the end of the treatment, statically significant changes were determined in Oswestry scale score and PF, BP, GH, and MH parameters (Table 1).

The comparison of parameters in Group 3 before the therapy and at the end of the therapy revealed significant changes is VAS, Oswestry scale score, and PF, RP, BP, GH, and RE parameters (*P* < 0.05). VT, SF, and MH parameters did not significantly change in Group 3 (*P* > 0.05). Comparing results at the end of the treatment and in third month of the treatment, statically significant change was continuing in Oswestry scale score and BP and GH parameters (Table 1).

Comparing the changes between groups, statically significant difference was observed in MH parameter before treatment between Groups 1 and 2 and in MH parameter and VAS score in third month of the therapy between Groups 2 and 3. However, the evaluation of the patients after ten days of treatment did not show significant differences between the groups compared to baseline values (Table 1).

## 4. Discussion

In the present study, a total of 65 patients with lumbar disc herniation in the high intensity laser treatment (HILT), US, and control groups were compared in terms of their scores in VAS, SF-36, and Oswestry scale. In all treatment groups, most parameters measured showed significant changes. The differences in the three treatment groups did not achieve statistical significance in terms of some parameters (*P* > 0.05). The comparison of parameters in Group 1 before and at the end of the therapy revealed significant changes in VAS score, Oswestry scale score, BP, GH, VT, and SF. The comparison of parameters in Group 2 before and at the end of the therapy revealed significant changes in VAS, Oswestry scale score, and PF, RF, BP, GH, VT, SF, RE, and MH parameters. The comparison of parameters in Group 3 before therapy and at the end of the therapy revealed significant changes in terms of VAS, Oswestry scale score, and PF, RP, BP, GH, and RE parameters. Improvement of Oswestry scale score and PF, BP, GH, and VT parameters in Group 1, improvement of Oswestry scale score and PF, BP, GH, and MH parameters in Group 2, and improvement of Oswestry scale score and BP and GH parameters in Group 3 were going on increasingly for three months. VAS scores were better than compared to value before the therapy and at the end of the therapy but there was no significant difference between VAS scores in third month after the therapy and at the end of the therapy.

Fiore et al. demonstrated the short term effects of a high intensity laser on lumbar pain in a study that included 30 patients, 15 of which received US therapy and 15 who received laser therapy. They reported more prominent pain relief and recovery disability in the HILT group compared to US group after three weeks of treatment. The rate of decline in the VAS score in the two patient groups was 10% in favor of the HILT group and 20% in Oswestry scale in favor of the HILT group. They did not have control group as an important lack of the study [13]. Alayat et al. conducted a randomized, single-blind, placebo-controlled study to evaluate the long term effects of HILT in patients with lumbar pain. The study included 72 patients, and 28 patients in Group 1 received HILT + exercise therapy, 24 patients in Group 2 received placebo laser + exercise, and 20 patients in Group 3 received HILT. They performed a total of 12 sessions of therapy for four weeks. The patients were evaluated at baseline, fourth week, and twelfth week. This study showed higher efficacy of the HILT + exercise program compared to placebo HILT + exercise program and only exercise group [14]. Conte et al. evaluated the HILT + lumbar school versus lumbar school alone and studied 28 patients using VAS and Oswestry scale. They emphasized that the HILT + lumbar school provided higher improvement in Oswestry and VAS scores compared to the lumbar school alone. Furthermore, they concluded that the laser possessed low biological activity and produced little side effects, if any, compared to pharmacological therapies [15]. A meta-analysis of studies on low intensity laser therapy reported positive effects on tissue repair and pain control at various levels. However, these studies did not specifically evaluate lumbar pain [16]. Monochromatic laser beams can inherently modulate cellular and tissue functions. There are controversial data regarding the effects of low intensity laser on lumbar pain. Despite this controversy, low intensity laser therapy has demonstrated efficacy in the short term compared to the placebo when the patients were assessed using VAS and Oswestry scales [17]. Considering last surveys, HILT therapy can be good alternative physical therapy agent for the patients with lumbar disc herniation. It does not have distinct adverse effect and we did not encounter any complication in our study.

Therapeutic US is an important treatment agent in musculoskeletal disorders [12]. Ebadi et al. evaluated a total of 50 patients divided into two groups in order to investigate the efficacy of continuous US in chronic lumbar pain. The first group received continuous US and exercise, and the second group received placebo US + exercise. They performed a total of ten sessions of therapy for four weeks. They evaluated the patients before and after the therapy using FRI (functional rating index), VAS score, ROM, and endurance time. They found significant improvement in the FRI index in the continuous US group. The decrease in VAS scores, increase in lumbar ROM, and endurance time were more prominent in the continuous US group compared to the placebo US group. The limitation of this study was that the effectiveness of placebo US was not evaluated with the addition of a third group that received exercise only [18]. Durmus et al. also evaluated three patients groups that received either US + exercise therapy, electrical stimulation, and exercise therapy or exercise therapy alone for lumbar pain. They found that US + exercise provided better pain relief compared to the other two treatment modalities [19]. Doğan et al. divided 60 patients into three groups in order to evaluate three different approaches in the treatment of chronic lumbar pain. In their study, Group 1 received home exercises + aerobic exercise, Group 2 received physical therapy (hot-pack, TENS, and US) and home exercises, and Group 3 received home exercises alone. They found a significant reduction in the pain level and an increase in aerobic capacity, but there was no significant difference between the groups. They stated that the rate of functional disability and physiological disturbances were lower in the physical therapy and home exercise group [20]. In the study by Grubisić et al. that evaluated the therapeutic efficiency of US in the treatment of chronic lumbar pain, 16 out of 31 patients received US therapy. Ongoing medical therapies of the study participants were not changed and the patients were only allowed to take paracetamol during painful periods. In the control group, a US device was switched off while performing physical therapy. At the end of the treatment period, US was found to be more effective in providing pain relief; however, US was not found to be superior to the control group in providing functional improvement [21]. Basford et al. reported that US therapy has gained a wide acceptance in routine practice in the treatment of chronic lumbar pain; however, the evidence is not strong enough to support the efficiency of this therapy [17]. US is used for a long time in physical therapy and it is so safe and effective treatment agent in several locomotor diseases. In our study, we obtained improvement in terms of some parameters in US group. We did not encounter any complication.

Limitation of this study may be the number of the patients. If we received a larger numbers of patients, the effect of HILT would be displayed obviously. We permitted the patients to take medicine only during treatment period. It may be seen disadvantage for the short term effect of the treatment. Further studies with a larger number of patients and controls are required to evaluate the long term effects of the therapies.

There were no studies in the literature that compared the effectiveness of HILT, US, and medical therapies in patients with lumbar disc problems, which have an important place in the etiology of acute and chronic lumbar pain. The current literature search did not show a sufficient number of similar studies. There are a very limited number of studies that evaluated the efficiency of HILT in lumbar pain. The number of patients included in the present study was similar to that reported in other studies in the literature. The inclusion of a control group allowed for the comparison of HILT and US therapies with exercise therapies in the short term. However, the evaluation of the patients aftermath ten-day treatment did not show significant differences between the groups compared to baseline values. This may have been caused by the fact that the patients in our study were allowed to take medical therapies during the most painful periods. The patients in the HILT and US groups were not allowed to take medical therapies unless they had extreme pain. We found that HILT, US, and exercise were efficient therapies for lumbar discopathy but HILT and US had longer effect in terms of some parameters. Exercise therapy should never be ignored to treat and prevent lumbar back pain.

## Figures and Tables

**Table 1:** Changes in VAS scores, Oswestry scale scores, and SF-36 parameters in the three groups before, at the end of treatment, and in third month after therapy.

	Laser group			Ultrasound group			Control group			*P* ^1^ value	*P* ^2^ value	*P* ^3^ value
	Pre-treat	Post-treat	3. month	Pre-treat	Post-treat	3. month	Pre-treat	Post-treat	3. month			
VAS	7.55	4.00	3.25	7.52	3.40	2.96	7.60	4.75	4.80	0.983	0.169	0.013^*^
OS	49.10	43.00	70.25	51.92	40.40	26.64	51.20	43.00	32.70	0.809	0.784	0.502
PF	41.25	45.75	71.25	35.80	48.60	66.40	33.25	46.00	60.75	0.335	0.874	0.431
RP	38.75	42.50	0.083	30.00	54.00	58.00	50.00	67.50	71.25	0.266	0.134	0.499
BP	25.75	38.85	63.60	24.36	36.92	60.12	21.00	33.15	54.50	0.661	0.369	0.507
GH	37.70	41.55	65.80	33.96	38.12	54.88	34.10	39.70	54.30	0.695	0.810	0.258
VT	44.00	47.85	63.50	47.60	53.40	62.20	46.00	50.50	49.50	0.492	0.333	0.107
SF	318.75	327.50	312.50	295.00	232.00	387.00	187.50	156.25	241.25	0.166	0.098	0.325
RE	208.30	185.00	250.05	94.64	191.96	244.00	196.60	188.35	183.40	0.119	0.995	0.653
MH	65.40	67.40	72.8	58.56	66.40	74.88	59.05	59.60	61.20	0.020^*^	0.074	0.035^*^

VAS: visual analog scale, OS: Oswestry scale score, PF: physical function, RP: restricted physical roles, BP: body pain, GH: general health, VT: vitality, SF: social functioning, RE: restricted emotional roles, and MH: mental health. Pre-treat: before treatment, Post-treat: after treatment, and 3. month: measures of the patients 3 months later after treatment. *P* ^1^: *P* value obtaining by which comparing statistically scores of the scales among the groups before treatment, *P* ^2^: *P* value obtaining by which comparing statistically scores of the scales among the groups after treatment, and *P* ^3^: *P* value obtaining by which comparing statistically groups scores of the scales 3 months later after treatment.

^*^VAS score is statistically significant between groups in third month after treatment.
